# Development of a user-friendly calculator for a pediatric split-bolus polytrauma computed tomography protocol

**DOI:** 10.1007/s00247-024-06082-5

**Published:** 2024-11-01

**Authors:** Ana Carolina Rocha, Leonor Alamo, Nemanja Ostojic, Christine Chevallier, Estelle Tenisch

**Affiliations:** University Hospital of Lausanne, Rue de Bugnon 21, CH-1011 Lausanne, Vaud Switzerland

**Keywords:** Child, Contrast media, Diagnostic imaging, Multidetector computed tomography, Multiple trauma, Radiation protection, Whole body imaging

## Abstract

We present here a user-friendly calculator for the setting of a pediatric split-bolus polytrauma computed tomography (CT) protocol with a mixed arterial and venous phase, aiming to both reduce radiation dose and improve workflow while assuring optimal image quality. All the different parameters are calculated based on patient’s weight with rapid computation of the injected contrast media and saline volumes, injection’s flow rate, injection’s timing, and optimal acquisition time. The designed calculator is built in a widely available Google Sheets file, accessible by a quick response (QR) code. Although polytrauma imaging represents the main goal of the technique, it can be used in a wide variety of contexts, including oncological, infectious, and vascular pathologies.

**Graphical Abstract:**

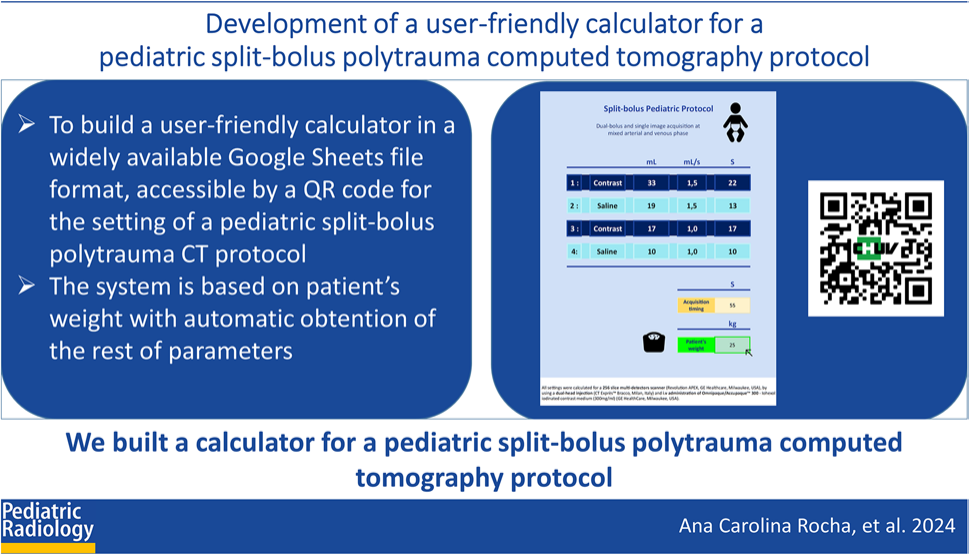

## Introduction

The split-bolus computed tomography (CT) technique consists of a single CT acquisition after two separate contrast media injections, resulting in combined opacification phases [1]. Its first clinical use was the study of the upper urinary tract through the acquisition of synchronous nephrographic and late renal excretory phase images [2]. Nowadays, the technique is gaining relevance in the context of the “as low as reasonably achievable” (ALARA) principle as it allows to reduce radiation dose while providing appropriate information in a single imaging acquisition with arterial and venous opacification along with parenchymal impregnation. Such acquisition technique is particularly interesting in a polytrauma context but can also be used in a wide spectrum of oncological, infectious, or vascular inquiries [3].

## Background

Success in obtaining a split-bolus CT acquisition with optimal arterial and venous opacification is challenging, especially in the pediatric population, a heterogeneous group with a wide variability of height and weight. The preparation of such radiological examinations is often a source of stress for all participants that can delay image acquisition. Moreover, the selection of inadequate injection parameters may limit the quality of images and consequently, the accuracy of the diagnosis [3]. This often leads non-pediatric centers to perform multiphase acquisitions as they would for adult polytrauma patients, resulting in an increased final radiation dose.

A review of literature shows that although multiple teams suggested different calculator methods for a split-bolus polytrauma CT protocol, most of them are difficult to apply in stressful situations because of the calculations they involve [4–6]. Therefore, we decided to construct a user-friendly protocol calculator and to make it widely available in Google Sheets (Google LLC, Mountain View, CA) format by a quick response (QR) code.

## Description of new technical innovation

All settings listed below were calculated for a 256-slice multidetector scanner (Revolution APEX, GE Healthcare, Milwaukee, WI), by using a dual-head injection (CT Exprès™ Bracco, Milan, Italy) and i.v. administration of Omnipaque/Accupaque™ 300—Iohexol iodinated contrast medium (300 mg/ml) (GE HealthCare, Milwaukee, WI).

The aim of this pediatric split-bolus protocol is to perform accurate arterial and venous opacification along with parenchymal impregnation, following two contrast media injections in a single CT acquisition. The first injection will provide venous opacification and parenchymal impregnation whereas the second injection will serve to opacify the arterial bed.

All different settings including acquisition time, contrast media and saline volumes, flow rate injection, injection time, and pause time between the two injections are calculated based exclusively on the patient weight in kilograms, which is the only information to insert in the calculator. Settings can be calculated for a weight range between 4 and 100 kg.

As suggested by Kim et al. [6], the total injected contrast media volume in milliliters is two times the patient weight for small children weighing ≤ 30 kg. The primary reasoning is that lighter patients have faster heart rates, smaller body sizes, and reduced blood volumes, resulting in quicker circulation. However, for patients > 30 kg, who have more adult-like physiology and blood pool, a volume of 30 ml is added to the patient weight, up to a maximum of 80 ml. However, contrary to Kim et al. [6], two-thirds of the so-obtained total contrast volume is injected in the first bolus (venous phase) and the remaining one-third in the second bolus (arterial phase). This adjustment ensures adequate parenchymal impregnation and venous opacification, which are more critical than arterial opacification following intravenous injection.

The timing of image acquisition (venous phase) is set to 50 s after the beginning of the first bolus for patients≤15 kg, 55 s for patients>15 kg and ≤ 30 kg, and 65 s for patients>30 kg of weight.

The timing of the second bolus (arterial phase) is set to 7 s before image acquisition for the patients weighting≤5 kg, 10 s for those>5 kg and ≤ 10 kg, 15 s for those10 kg and ≤ 18 kg, and 20 s for those > 18 kg of weight.

Between the two boluses, a calculated pause is set for the patients ≤ 15 kg and a saline flush for the others, with their duration resulting from the time interval remaining between the two contrast media boluses. The saline volume results from the rate of injection allowed by the patient weight, with a minimum volume set at 10 ml, which is the volume of the tubing of the pump. After the two contrast media injections, a saline flush with 5 ml is set for patients ≤ 15 kg and with 20 ml for those > 15 kg.

The flow rate of each contrast media bolus and saline flush was set based on five weight categories (Table 1). The total fluid load, of contrast media and saline, was limited to 3 ml/kg of patient weight.

**Table 1:** Contrast media bolus and saline flush injection flow rates based on weight categories

Weight (kg)	Bolus 1 (ml/s)	Saline 1 (ml/s)	Bolus 2 (ml/s)	Saline 2 (ml/s)
< 10	0.6	0 (pause)	0.6	0.5
≥ 10 < 16	1	0 (pause)	0.8	0.6
≥ 16 < 18	1	1	0.8	0.6
≥ 18 < 30	1.5	1.5	1	1
≥ 30 < 40	1.7	1.5	1.5	1.5
≥ 40 < 100	2	2	2	2

As the timings are calculated from the start of the first contrast media injection, the tubing must be purged with contrast media for all patients.

**Fig. 1:**
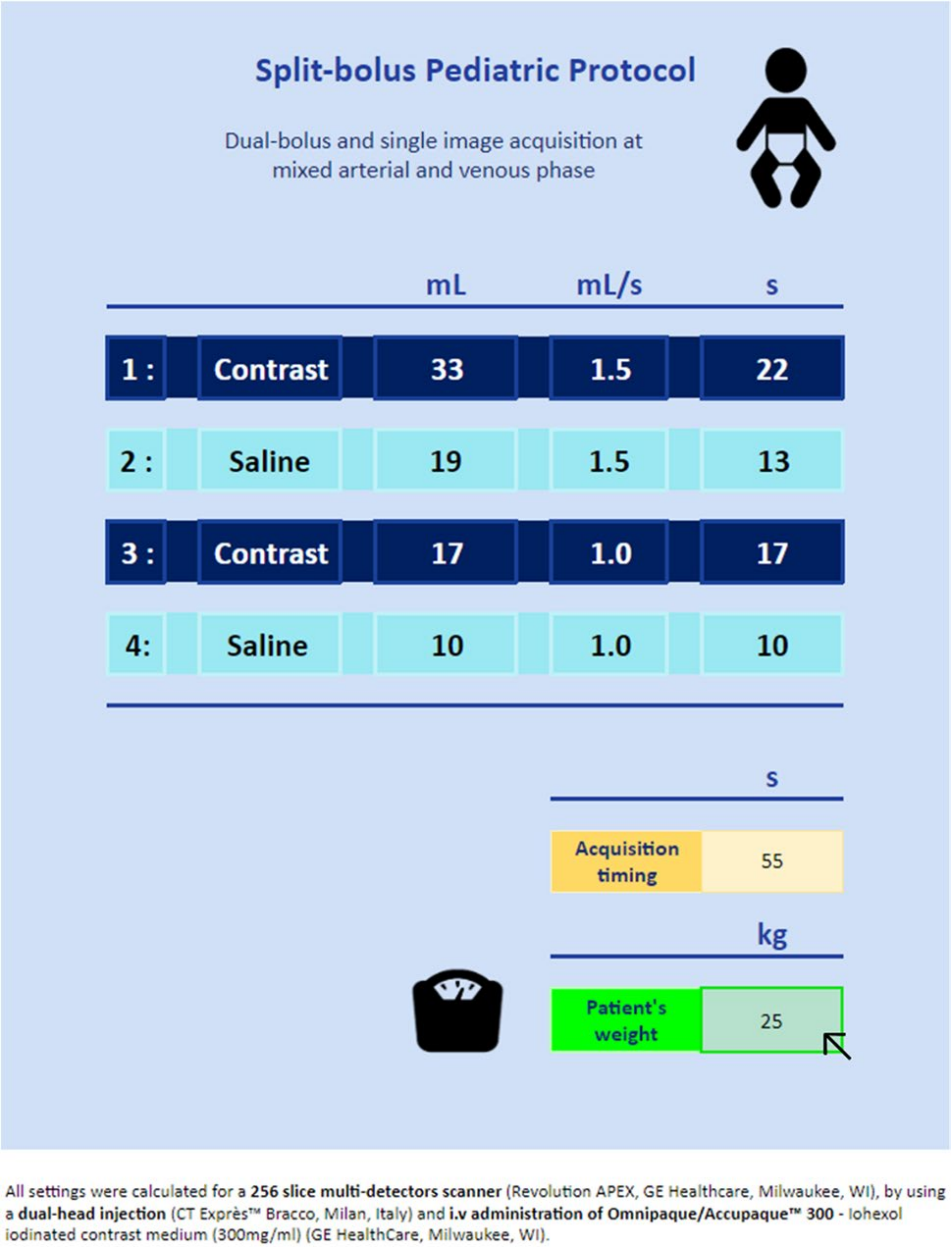
Calculator display. The only modifiable cell is the light green one where the patient’s weight is inserted in kg (*arrow*) and automatically all injection settings and acquisition timings are presented

**Fig. 2:**
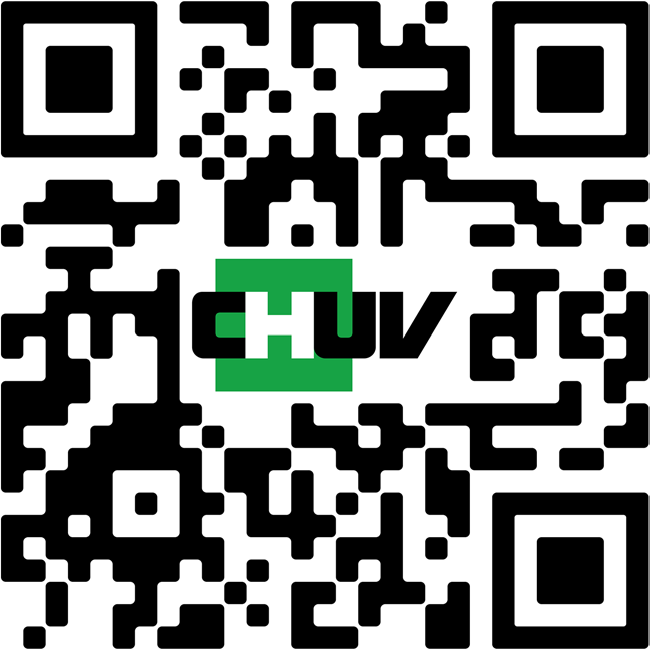
Quick response (QR) code leading to the Google Sheets with the calculator

The calculator following the previously described rules was built in a Google Sheet table, to ensure file compatibility and transmission. To facilitate its use, we chose a similar design to the display of the dual-headed injector (CT Exprès™ Bracco, Milan, Italy) used in our department (Fig. 1). The calculator is accessible by a QR code link (Fig. 2).

## Discussion

**Fig. 3:**
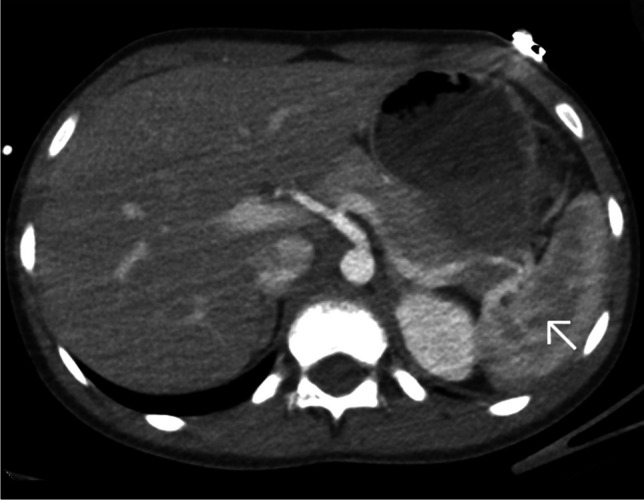
A 10-year-old girl with a history of a ski fall. Axial computed tomography, post-contrast, following a split-bolus protocol upper-abdominal image (soft tissue window). A too early acquisition resulted in suboptimal image quality with an inhomogeneous splenic enhancement (*arrow*), with alternating hypodense stripes mimicking splenic lacerations

The split-bolus technique allows an important reduction in exposure while providing accurate information for both arterial and venous vessels as well as for parenchymal evaluation, providing significant advantages, especially in pediatric patients. In our department, this tool was introduced in June 2023, but both the medical team and the radiographers found some difficulties for a rapid assessment of the multiple parameters required for the exam, which in some cases resulted in a delay in the preparation of the exam, inadequate selection of settings, and limited quality of the CT-exam (Fig. 3).

The calculator presented here was introduced in our hospital and has been regularly used since February 2024, with excellent results. Both the medical team and radiographers find the procedure easy and reliable to use, which has contributed to reduce the stress of preparing the exam and decreased imaging delays but above all, to minimize the risk of errors.

**Fig. 4:**
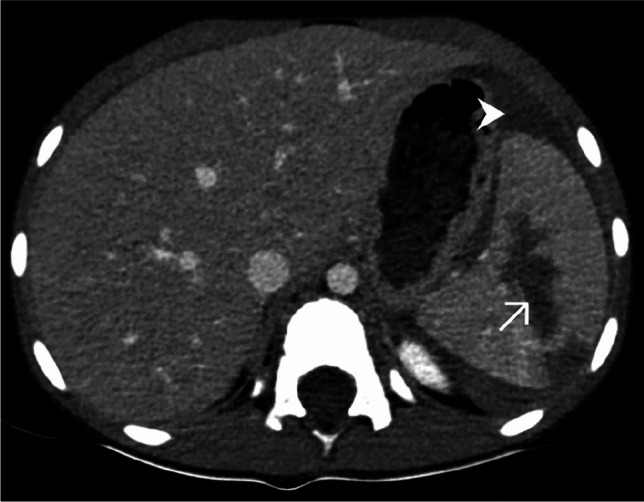
A 5-year-old boy with a history of a ski fall. Axial computed tomography, post-contrast, following a split-bolus protocol, upper-abdominal image (soft tissue window). American Association for the Surgery of Trauma (AAST) grade III splenic laceration seen as a hypodense irregular stripe (*arrow*) and peri-splenic fluid (*arrowhead*)

**Fig. 5:**
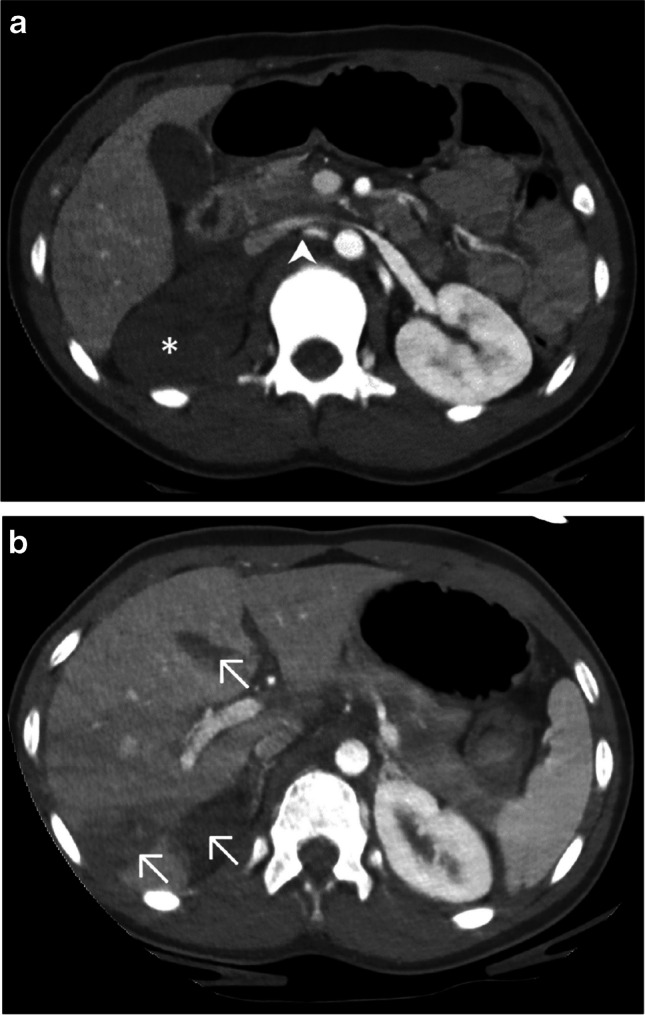
A 10-year-old girl with a history of a 20-m fall. Axial CT, post-contrast, following a split-bolus protocol, upper-abdominal images (soft tissue window). **a** Right renal infarction seen as a complete absence of enhancement of the kidney (*asterisk*) after renal artery dissection seen as a truncated arterial course (*arrowhead*). **b** American Association for the Surgery of Trauma (AAST) grade III hepatic lacerations seen as hypodense irregular stripes (*arrows*)

After a polytrauma event that requires a complete head, neck, and trunk evaluation, this technique can be incorporated following a non-enhanced brain-CT, allowing to perform a single acquisition from the vertex to the pelvis. In our experience, visceral parenchymal lesions and most vascular traumatic lesions are easily depicted (Fig. 4 and Fig. 5). However, although the indication remains quite rare in the pediatric population, caution must be taken in the specific search for a pseudoaneurysm as it can be masked by venous opacification [7].

**Fig. 6:**
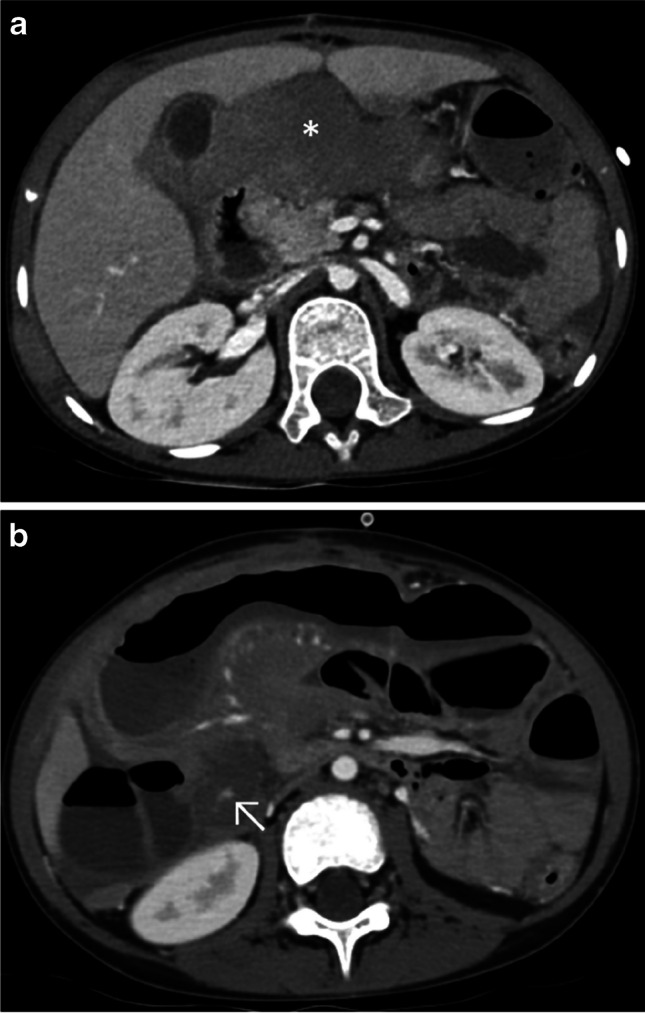
A 7-year-old boy with a history of a rapidly progressive Burkitt lymphoma. Axial computed tomography, post-contrast, following a split-bolus protocol, upper-abdominal images (soft tissue window). **a** Tumoral mass seen as a hypo- to isodense space-occupying lesion (*asterisk*). **b** Oncological complication with active intestinal hemorrhage, seen as hyperdense foci indicating extravasation of contrast media (*arrow*)

**Fig. 7:**
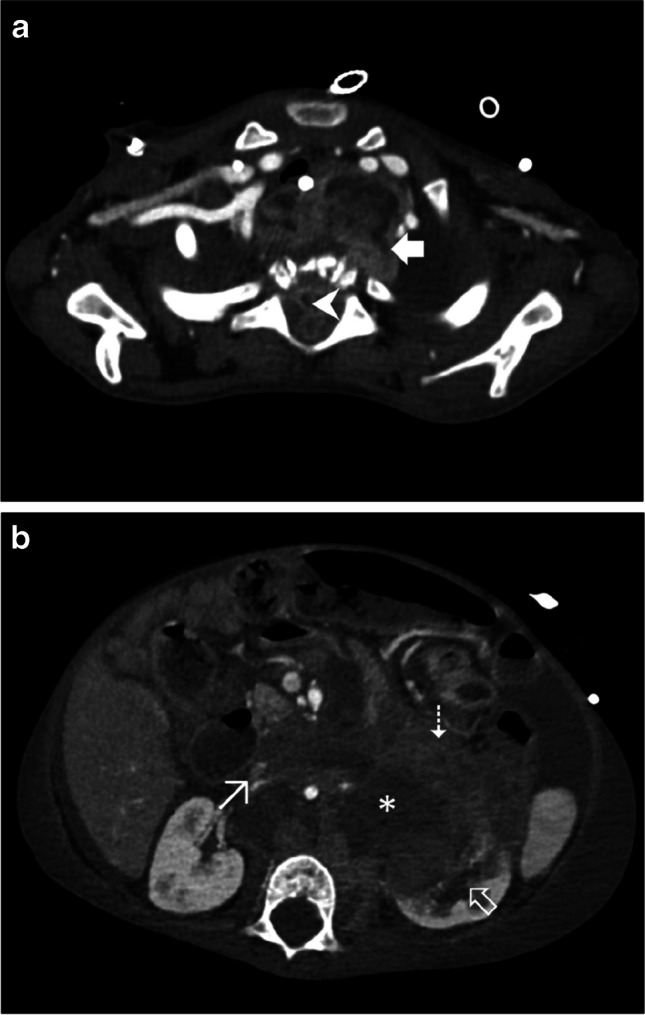
A 1-year-old boy with a history of an extensive thoracic and abdominal neuroblastoma. **a** Axial computed tomography (CT), post-contrast, following a split-bolus protocol, upper-thoracic image (soft tissue window) with tumoral mass (*solid bold arrow*) in the mediastinum invading the spine canal (*arrowhead*). **b** Axial CT, post-contrast, following a split-bolus protocol, upper-abdominal image (soft tissue window) with mass (*asterisk*) in the retroperitoneum invading the left kidney (*outlined bold arrow*) and the pancreatic tail (*dashed arrow*). Note the laminated aspect of the inferior vena cava (*thin arrow*)

We have recently incorporated this protocol for some selected oncological evaluations (Fig. 6 and Fig. 7), with promising first results [8].

## Data Availability

No datasets were generated or analysed during the current study.
